# Nutrition in early life, epigenetics and lifelong health – evidence from cohort and intervention studies

**DOI:** 10.1017/S0029665125102061

**Published:** 2025-12-26

**Authors:** Keith Godfrey, Paula Costello, Sarah El-Heis

**Affiliations:** 1 MRC Lifecourse Epidemiology Centre, University of Southampton; 2 NIHR Southampton Biomedical Research Centre, University of Southampton and University Hospital Southampton NHS Foundation Trust

**Keywords:** nutrition, early life environment, epigenetics

## Abstract

This review summarizes evidence from cohort and intervention studies on the relationships between nutrition in early life, epigenetics, and lifelong health. Established links include maternal diet quality with conception rates, micronutrient sufficiency before and during pregnancy with preterm birth prevention, gestational vitamin D intake with offspring bone health, preconception iodine status with child IQ, adiposity with offspring obesity, and maternal stress with childhood atopic eczema. Animal studies demonstrate that early-life environmental exposures induce lasting phenotypic changes via epigenetic mechanisms, including DNA methylation, histone modifications, and non-coding RNAs, with DNA methylation of non-imprinted genes most extensively studied. Human data show that nutrition during pregnancy induces epigenetic changes associated with childhood obesity risk, such as Antisense long Non-coding RNA in the INK4 Locus (ANRIL, a long non-coding RNA) methylation variations linked to obesity and replicated across multiple populations. Emerging insights reveal that paternal nutrition and lifestyle also modify sperm epigenomics and influence offspring development. Although nutritional randomised trials in pregnancy remain limited, findings from the NiPPeR trial showed widespread preconception micronutrient deficiencies and indicated that maternal preconception and pregnancy nutritional supplementation can reduce preterm birth and early childhood obesity. The randomised trials UPBEAT and MAVIDOS have shown that nutritional intervention can impact offspring epigenetics. Postnatal nutritional exposures further influence offspring epigenetic profiles, exemplified by ALSPAC cohort findings linking rapid infant weight gain to later methylation changes and increased obesity risk. Together, these studies support a persistent impact of maternal and early-life nutrition on child health and development, underpinned by modifiable epigenetic processes.

## Introduction

Nutrition in early life plays a crucial role in shaping lifelong health outcomes. The early life period, spanning from conception to early childhood, is a critical window of susceptibility during which environmental factors can influence developmental processes and establish a trajectory of health or disease^(1)^. Nutrition, including micronutrient status and body composition, is foremost among such environment factors, which also include exposure to parental smoking, endocrine disrupting chemicals, biological and psychosocial stress. The concept of developmental origins of health and disease (DOHaD) proposed that early life exposures can have lasting impacts on health and disease risk later in life^(1)^. The DOHaD concept emerged from epidemiological studies in the UK during the 1980s, which revealed a clear relationship between lower birth weight and an increased risk of death from non-communicable diseases (NCDs) in adulthood, including cardiovascular disease^(2)^ and chronic obstructive airways disease^(3)^. Impaired fetal development has been linked to a higher risk of a range of adult NCDs that increase premature mortality risk, including type 2 diabetes, metabolic syndrome, osteoporosis, sarcopenia, and coronary heart disease^(4)^, and to negative outcomes in childhood, such as stunted growth and reduced cognitive function^(4,5)^. These associations have been consistently replicated and are recognised as being exacerbated by environmental risk factors encountered in the postnatal environment.

To date, fixed genomic variations such as single nucleotide polymorphisms and copy number variations have been found to explain only a modest proportion of the risk associated with NCDs and it is increasingly believed that the developmental environment plays a significant role in shaping later-life phenotypes by modifying epigenetic regulation of genes^(6)^. Epigenetic processes including DNA methylation, histone modifications and non-coding RNAs regulate gene expression by modulating the packaging and expression of the DNA without a change in genomic sequence. These changes can be maintained over multiple divisions in somatic cells. Environmental and lifestyle influences such as nutrition and stress can induce epigenetic modifications, effectively making the epigenome a molecular record holding the “memory” of past exposures. During development, epigenetic processes contribute to phenotypic plasticity, allowing the fetus to adapt to predicted postnatal environments^(7)^. However, when the phenotype is mismatched to the later environment, e.g. from inaccurate nutritional cues from the mother or placenta or rapid socioeconomic shifts, this “mismatch” can increase the risk of NCDs in adulthood^(8)^.

The impact of developmental epigenetic changes may not become apparent until later in life. Epigenetic biomarkers may therefore serve as indicators of previously undocumented developmental exposures and as predictors of future disease risk, enabling early intervention strategies to improve both early development and later health. Discovery and validation of perinatal epigenetic biomarkers, with both replication in independent cohorts and in vitro validation, is therefore an important and growing field. Recent advances in enzymatic approaches and high-throughput sequencing have enabled epigenetic biomarker discovery on a genome-wide scale^(9)^. The expanded coverage provided by these platforms is likely to uncover many new disease-related epigenetic modifications located outside well-known candidate regions such as CpG islands and gene promoters, complementing traditional candidate gene and array-based approaches that only interrogate small regions of the genome.

Epigenetic changes can be tissue specific and can have tissue specific consequences. Skeletal muscle has a lower priority in nutrient partitioning in the developing fetus, compared with the brain and heart, thus making it particularly vulnerable to nutrient deficiency. Epidemiological studies have consistently linked negative early-life environmental exposures to reduced muscle mass and function in later life^(10–12)^, with epigenetic changes proposed as mediators. Recent research measuring CpG methylation across the genome (the “methylome”) of cultured myoblasts isolated from older individuals has shown associations with birthweight, growth during infancy, and childhood illnesses^(13)^, with some of the differentially mediated regions associated with later-life grip strength and sarcopenia. In this review, we will summarize the evidence from cohort and intervention studies on the relationships between nutrition in early life, epigenetics, and lifelong health.

### Maternal Diet and Fetal Development

A mother’s preconception and pregnancy diet, micronutrient status, body composition, metabolism, mood and lifestyle are all implicated in maternal pregnancy outcomes and offspring body composition, cardiometabolic, neurobehavioural and allergic outcomes. Examples include maternal diet quality and conception rates^(14)^, micronutrient intake and preterm birth^(15)^, vitamin D supplementation and infantile atopic eczema^(16)^, iodine status and child IQ^(17)^, maternal adiposity and offspring obesity^(18)^, and maternal stress and offspring atopic eczema^(19)^.

Maternal diet during pregnancy is a critical determinant of fetal development and has been linked to a range of health outcomes in offspring^(20–22)^. A maternal diet rich in fruits, vegetables, and whole grains, has been associated with improved fetal growth and development, as well as a reduced risk of adverse birth outcomes, such as preterm birth and low birth weight^(23)^. In contrast, maternal diets high in sugar, salt, and unhealthy fats, has been linked to an increased risk of adverse outcomes^(24)^, and maternal diets with a high inflammatory potential have been associated with increased offspring adiposity during childhood^(25)^.

The Growing Up in Singapore Towards healthy Outcomes (GUSTO) cohort study, a prospective mother-offspring cohort study, has made significant contributions to our understanding of the relationships between maternal diet, fetal development, and lifelong health^(25)^. In this study, maternal diet was assessed using a 24-hour recall and food diary at 26-28 weeks of gestation^(26)^. Associations were identified between a high-quality dietary pattern during pregnancy and lower risks of preterm birth and excessive offspring adiposity during childhood^(27,28)^. The findings of this study have highlighted the importance of a healthy maternal diet in promoting optimal fetal growth and development.

### Epigenetics and Early Life Nutrition

Epigenetics encompasses a variety of heritable modifications that regulate gene expression without altering the underlying DNA sequence. These modifications can be stably maintained through multiple somatic cell divisions, thereby contributing to long-term gene regulation. The most widely studied form of DNA methylation is where a methyl group is added to the cytosine base of a CpG dinucleotide, primarily to regulate gene expression. This methylation can alter gene expression by either directly blocking the binding of transcription factors to DNA or by recruiting repressive protein complexes that induce local chromatin remodelling^(29)^. Patterns of DNA methylation are usually studied within CpG rich islands in gene promoter regions^(30)^, but gene body and intergenic region DNA methylation are also thought to influence cell physiology. Histone modifications such as acetylation, methylation, ubiquitination and phosphorylation can directly affect chromatin structure and therefore the accessibility of the underlying genomic sequence, while also providing binding sites for proteins involved in gene regulation. Other mechanisms include the non-coding RNAs (ncRNA), which are functional RNA molecules that are not translated into proteins. They can mediate mRNA degradation or translational repression and, when targeted to the promoter region of a gene, induce both DNA methylation and repressive histone modifications^(31)^.

Studies in animal models have shown that maternal diet can induce lasting metabolic changes in offspring by modifying the epigenetic regulation of key metabolic genes. For example, when pregnant rats were fed a protein-restricted diet, their offspring showed reduced DNA methylation of the glucocorticoid receptor (GR) and peroxisome proliferator-activated receptor alpha (PPARα) genes in the liver^(32)^. This epigenetic change was associated with increased expression of GR and PPARα and a persistent change in metabolic pathways, specifically enhanced gluconeogenesis and fatty acid β-oxidation, which are regulated by these nuclear receptors. With growing concern over the widespread consumption of energy-dense Western diets, many studies have turned their attention to the effects of maternal high-fat intake. In rats, maternal high-fat feeding during pregnancy has been shown to reduce expression of FADS2, the rate-limiting enzyme in polyunsaturated fatty acid synthesis in the liver of offspring, and this reduction is accompanied by altered DNA methylation at CpG sites within the gene’s promoter region^(33)^. Similarly, in mice, maternal obesity and diabetes have been linked to widespread changes in DNA methylation in the liver of offspring^(34)^. Notably, the window of epigenetic plasticity may extend beyond the prenatal period into postnatal life. For example, overfeeding in rat pups led to hypermethylation at two CpG sites in the promoter of proopiomelanocortin (POMC), a gene critical for appetite regulation. This hypermethylation prevented the upregulation of POMC expression in response to elevated plasma leptin and insulin levels^(35)^.

### Evidence from human cohort studies

Evidence from human cohort studies suggests a similar important role for epigenetic processes in holding the “memory” of developmental exposures, with long-term consequences for the risk of NCDs. For example, these have linked nutritional exposures during pregnancy to epigenetic changes that increase offspring susceptibility to childhood obesity^(36)^.

The prevalence of childhood obesity is rising rapidly, posing immediate health risks for children and increasing their likelihood of developing obesity and related metabolic disorders in adulthood ^(37)^. The National Child Measurement Programme (NCMP) has also shown the disparity gap in child obesity widening each year, mostly driven by rising obesity rates in the most deprived areas and a relatively stable prevalence among the least deprived children. The gap in obesity prevalence between the most and least deprived areas in 2022-23 has reduced compared to 2020-21 from 19.5 to 17.1 percentage points, but is still much larger than that seen in pre-pandemic years^(38)^.

Several early-life risk factors have been identified that significantly increase the likelihood of childhood obesity. In the prospective Southampton Women’s Survey (SWS) parent-offspring cohort, five key risk factors were defined: maternal obesity, excessive gestational weight gain, low maternal vitamin D levels, smoking during pregnancy, and a short duration of breastfeeding. At both 4 and 6 years of age, there was a positive graded association between the number of these early-life risk factors and increased childhood adiposity and obesity. After adjusting for potential confounders, children exposed to four or five risk factors had a relative risk of being overweight or obese of 3.99 (95% confidence interval (CI): 1.83–8.67) at age 4, and 4.65 (95% CI: 2.29–9.43) at age 6, compared to children with no risk factors^(39)^. Similarly, in the GUSTO prospective cohort study six key risk factors were examined: maternal pre-pregnancy overweight/obesity, paternal overweight/obesity at 24 months post-delivery, excessive gestational weight gain, raised maternal fasting glucose during pregnancy (≥5.1 mmol/L), breastfeeding duration <4 months and early introduction of solid foods (<4 months). The adjusted relative risk of overweight/obesity in children with four or more risk factors was 11.1 (95% CI: 2.5-49.1) at age 4, compared to children with no risk factors^(40)^. Early interventions to change these modifiable risk factors could therefore make a significant contribution to the prevention of childhood obesity. These findings have paved the way for a new series of systematic genome-wide epigenetic investigations to find epigenetic biomarkers associated with child adiposity. Higher methylation of the Retinoid X Receptor Alpha (*RXRA*) gene promoter at birth was associated with child’s later adiposity and associations were also observed between levels of RXRA methylation and mothers’ carbohydrate intake^(36)^. Further observational studies have characterised perinatal DNA methylation variations related to Antisense long Non-coding RNA in the INK4 Locus (ANRIL) that mark obesity risk, replicated across three populations and with relevant physiological effects of altering ANRIL methylation in vitro^(41)^. Decreased methylation of the *SLC6A4* promotor region, a transport protein responsible for reuptake of serotonin and which may play a role in appetite and energy balance, was associated with higher maternal gestational weight gain as well as increased adiposity in infancy, early childhood and adolescence^(42)^. Additionally, obese adults had lower methylation levels and decreased gene expression in adipose tissue compared to lean individuals^(42)^. These findings suggest that altered *SLC6A4* promoter methylation may provide a consistent marker of adiposity throughout the life course.

In the GUSTO cohort study, associations of maternal nutrition with pregnancy outcomes, fetal growth and childhood outcomes were examined, and DNA methylation assessed at birth. Dietary patterns rich in vegetables, fruits, and white rice were associated with lower risk of preterm birth and larger birth size, suggesting beneficial effects on developmental and growth outcomes^(27)^. Associations between low quality maternal diet and night-eating behaviours with higher insulin in the offspring were demonstrated and interact synergistically, especially in boys^(43)^. Additionally, in epigenome-wide association studies, higher maternal dietary glycaemic index and glycaemic load in pregnancy were associated with offspring cord blood DNA methylation at multiple CpG sites, with some relating to genes expressed in tissue relevant to metabolic health; associations were seen particularly in overweight and obese mothers^(44)^. The findings of this study highlighted the importance of maternal nutrition in shaping the epigenetic landscape of the newborn and suggested that these early life epigenetic modifications may have lasting impacts on health and disease risk later in life.

Emerging evidence supports a further influence of postnatal nutritional status on epigenetic processes. For example, a study in the ALSPAC cohort identified associations between rapid weight gain in infancy and small increases in childhood methylation at two CpG sites, one of which was replicated in the Southampton Women’s Survey and was also associated with subsequent overweight and obesity^(45)^.

### The importance of preconception health

Epidemiological, clinical and basic science research has identified the period around conception as being critical in the processes mediating parental influences on the next generation’s phenotype and health. During this time, from the maturation of gametes through to early embryonic development, the nutrition of mothers and fathers can adversely influence the offspring’s long-term risks of cardiovascular, metabolic, immune and neurological morbidities. Such ‘developmental programming’ has been demonstrated for exposures including maternal overnutrition and obesity; maternal undernutrition; related paternal factors; and from the use of assisted reproductive treatment^(46)^. Human studies and animal models demonstrate the underlying biological mechanisms, including epigenetic, cellular, physiological and metabolic processes.

### Paternal influences on epigenetic processes

While the main focus has been on mechanisms driven by maternal nutrition and other exposures, emerging evidence suggests that paternal nutrition and lifestyle also influence sperm epigenomics and transcriptomics, with consequences for the development of the offspring^(46,47)^. Meta-analysis of mouse paternal and maternal protein undernutrition indicate distinct parental periconceptional contributions to postnatal outcomes^(46)^. A 6-week paternal dietary intervention modified the small non-coding RNA (sncRNA) profile of human sperm in a subset of participants from the PREPARE trial^(47)^. sncRNA sequencing revealed that supplementation with olive oil, vitamin D, and omega-3 fatty acids altered the expression of 3 tRFs, 15 miRNAs, and 112 piRNAs, which target genes related to fatty acid metabolism and transposable elements in the sperm genome.

### Evidence from human intervention studies

While nutritional randomised controlled trials before and during pregnancy with offspring phenotyping are sparse, several such trials have reported findings of potential importance. For example, the UK Pregnancies Better Eating and Activity Trial (UPBEAT) of a nutritional intervention in women living with obesity^(48)^ showed that a low glycemic behavioural intervention can indeed change epigenetic processes in the offspring, with potential effects on adiposity in infancy. A pregnancy low GI diet reduced infant subscapular skinfold thickness at age 6 months by 0.26 SD and this beneficial effect of a prenatal nutritional intervention was also dependent on breastfeeding for ≥3 months^(49)^. Maternal GDM, as well as fasting, 1-hour, and 2-hour glucose levels from the OGTT, were associated with numerous differentially methylated CpG sites in the infant’s cord blood DNA. Notably, the methylation changes linked to GDM and 1-hour glucose were attenuated by the lifestyle interventions during pregnancy^(48)^.

Maternal vitamin D insufficiency is highly prevalent in many populations worldwide, and this can alter fetal bone growth and have lasting effects on the child’s bone health. In vitamin D insufficient mothers, splaying of the metaphysis (similar to childhood rickets) is present as early as 19 weeks of gestation^(50)^ and reduced concentration of 25(OH)-vitamin D in mothers during late pregnancy is associated with lower childhood bone-mineral content at age 9 years^(51)^.

Subsequent to the above observational studies, in the MAVIDOS trial supplementation of 1000 IU/day cholecalciferol during pregnancy did not affect the primary outcome of offspring neonatal bone mineral content (BMC), but did show a significant increase in infant bone mass for winter births^(52)^. In the same cohort, effects of gestational supplementation became more apparent over time; the intervention was positively associated with bone mineral density (BMD) at age 4 years^(53)^ and BMC and BMD at age 6-7 years^(54)^, suggesting a sustained beneficial effect of supplemental vitamin D supplementation in pregnancy on offspring bone health. Furthermore, significantly reduced DNA methylation at several CpG sites near the RXRA gene, known to play a role in bone metabolism, was observed in umbilical cord DNA^(55)^, suggesting a potential epigenetic mechanism by which maternal vitamin D supplementation may influence fetal bone development. Supplementation with 1000 IU/day cholecalciferol did not reduce the incidence of preterm birth but was associated with a greater likelihood of a spontaneous vaginal delivery^(56)^. MAVIDOS also provided the first randomised controlled trial evidence for the role of antenatal vitamin D supplementation in reducing the risk of infantile atopic eczema. The protective effects were seen in infants who breastfed more than one month but not in those who breastfed less than one month^(16)^.

In our multicentre NiPPeR randomised controlled trial we have reported that a maternal nutritional supplement taken preconception and during pregnancy substantially decreased the incidence of preterm birth, particularly cases associated with preterm pre-labour rupture of membranes^(57)^. Moreover, nutritional intervention before and during pregnancy halved the incidence of obesity in the offspring at age 2 years^(58)^. At recruitment preconception, over 90% of the trial participants had marginal or low concentrations of one or more of folate, riboflavin, vitamin B12, or vitamin D during, and many developed markers of vitamin B6 deficiency in late pregnancy^(59)^. Current work is examining the influence of the nutritional intervention on offspring epigenetics in the NiPPeR trial.

Evidence now points to the opportunity to reduce maternity disparities through intervention before and between pregnancies. In 2024 the NIHR launched its first ‘Challenge’ funding call, focused on new ways to tackle inequalities in maternity care. The resulting NIHR Maternity Disparities Consortium (2025-2030) will bring together a diverse range of organisations, including 9 lead UK Universities who will collaborate with local councils, NHS trusts, charities, industry and other health organisations. The Consortium will focus on inequalities before, during, and after pregnancy^(60)^.

## Conclusion

In conclusion, the evidence from cohort and intervention studies highlights the critical importance of nutrition in early life for shaping lifelong health outcomes. Maternal diet during pregnancy is a key determinant of fetal development and has lasting impacts on health and disease risk later in life. Epigenetics is a key mechanism by which early life nutrition can influence lifelong health, and interventions during early life have been shown to improve health outcomes later in life. Further research is needed to fully understand the relationships between nutrition in early life, epigenetics, and lifelong health, and to develop effective interventions to promote optimal health outcomes.

Collectively, the evidence supports lasting effects of maternal and infant nutrition on offspring health and human potential, with epigenetic processes likely to be an important underpinning mechanism.
